# The Emerging Utility of Bioimpedance in Patients with Chronic Obstructive Pulmonary Disease

**DOI:** 10.3390/medicina62040717

**Published:** 2026-04-09

**Authors:** Loredana-Crista Tiucă, Ninel Iacobus Antonie, Gina Gheorghe, Vlad-Alexandru Ionescu, Camelia Cristina Diaconu

**Affiliations:** 1 Faculty of Medicine, University of Medicine and Pharmacy Carol Davila, 050474 Bucharest, Romania; ninel-iacobus.antonie@drd.umfcd.ro (N.I.A.); gheorghe_gina2000@yahoo.com (G.G.); vladalexandru.ionescu92@gmail.com (V.-A.I.); camelia.diaconu@umfcd.ro (C.C.D.); 2 Internal Medicine Department, Clinical Emergency Hospital of Bucharest, 105402 Bucharest, Romania; 3 Section of Medical Sciences, Academy of Romanian Scientists, 050085 Bucharest, Romania

**Keywords:** bioelectrical impedance analysis, multifrequency bioimpedance analysis, chronic obstructive pulmonary disease, multimorbidity, body composition

## Abstract

*Background and Objectives*: Chronic obstructive pulmonary disease (COPD) is a major cause of morbidity and mortality worldwide and is frequently associated with multiple comorbidities. Bioelectrical impedance analysis (BIA) provides additional information on body composition and may contribute to the multidimensional assessment of patients with COPD. This study aimed to explore the relationship between BIA-derived parameters and clinical characteristics in hospitalized patients with COPD. *Materials and Methods*: A cross-sectional analysis of baseline data from a prospective cohort was conducted. A total of 72 hospitalized patients with COPD were included, according to predefined inclusion and exclusion criteria. All patients underwent multifrequency BIA using the InBody 380 device. Sociodemographic, clinical, and paraclinical data were collected and analyzed in relation to BIA-derived parameters. *Results*: Among the bioimpedance-derived parameters, phase angle (PhA) showed a significant correlation with clinical indices of disease severity, including the body mass index, airflow obstruction, dyspnea, and exercise capacity (BODE) index and the modified Medical Research Council (mMRC) dyspnea scale. Hydration-related parameters reflecting extracellular fluid distribution were associated with the presence of heart failure as a comorbidity. In addition, the evaluation of body fat using bioimpedance identified a higher number of patients with excess body fat compared with obesity defined according to the classical body mass index–based criteria. *Conclusions*: BIA may provide clinically relevant information on body composition and fluid distribution in patients with COPD. These findings support the potential role of BIA as a complementary tool in the multidimensional evaluation of multimorbid patients with COPD, although further studies are needed to clarify its prognostic value and clinical applicability.

## 1. Introduction

Chronic obstructive pulmonary disease (COPD) represents a major cause of morbidity and mortality worldwide, with a steadily increasing prevalence [1]. Despite its global impact, the true burden of COPD is likely underestimated, as a substantial proportion of affected individuals remain undiagnosed [2]. Population-based studies have reported that up to 70–80% of individuals fulfilling spirometric criteria for COPD are unaware of their condition, highlighting a significant gap in early detection and disease management [3].

COPD is currently recognized as one of the three leading causes of death globally, accounting for approximately 5% of all deaths worldwide in 2021, according to estimates reported by the World Health Organization (WHO) [1,4]. Notably, nearly 90% of COPD-related deaths occurring before the age of 70 years are reported in low- and middle-income countries, reflecting substantial disparities in access to healthcare, early diagnosis, and long-term disease management [1].

COPD is increasingly recognized as a condition associated with systemic inflammation, which contributes to the development of multiple extrapulmonary complications. Beyond respiratory impairment, this systemic inflammatory milieu plays a central role in the complex clinical phenotype of COPD and negatively influences disease progression and prognosis.

Body composition has emerged as an important prognostic determinant in patients with COPD. Malnutrition and sarcopenia are markers of disease severity and adverse outcomes [5,6]. Increasing attention has also been directed toward the concept of sarcopenic obesity, characterized by excess adiposity accompanied by sarcopenia. Although obesity is traditionally defined by body mass index (BMI) ≥ 30 kg/m^2^, a recently published consensus (2025) proposes a redefinition centered on excess adiposity, which may be assessed using several body composition techniques, including bioelectrical impedance analysis (BIA) and the measurement of percentage of body fat (PBF) [7]. This evolving framework opens new perspectives for the clinical application of body composition assessment, with potential implications for the multidimensional evaluation of patients with chronic diseases, including COPD.

In addition to alterations in muscle and fat compartments, changes in body water distribution between the extracellular and intracellular spaces have gained increasing attention. An expansion of the extracellular water compartment may facilitate the early detection of volume overload, potentially identifying patients at risk before overt clinical manifestations, such as peripheral edema, become apparent. Such changes may also signal the need for further diagnostic evaluation aimed at uncovering underlying cardiovascular or systemic contributors to fluid imbalance.

An accurate assessment of body composition remains challenging in routine clinical practice. Although several reference methods are available, including dual-energy X-ray absorptiometry (DXA) and deuterium dilution techniques, their use is limited by high costs, limited availability, technical complexity, and reduced feasibility in acutely ill or hospitalized patients. Other validated techniques, such as computed tomography or magnetic resonance imaging, while highly accurate, are similarly constrained by cost, radiation exposure, or logistical limitations, rendering them unsuitable for repeated or bedside assessments [8].

BIA has emerged as an attractive alternative, offering a non-invasive, rapid, and patient-safe method for evaluating body composition. The technique is easy to perform, requires minimal operator training, and can be applied at the bedside, making it particularly appealing in hospitalized populations. However, BIA also has important limitations. The method is not yet fully validated for routine clinical decision making across all patient populations, and results may vary depending on device-specific algorithms and proprietary software, which can limit comparability between different instruments and studies [8,9].

Despite these limitations, interest in BIA has increased substantially in recent years due to its practicality and repeatability. This has stimulated growing interest in exploring its potential utility in complex systemic conditions characterized by altered body composition, such as COPD [9,10].

Despite the growing body of evidence supporting the prognostic relevance of body composition and BIA-derived parameters in COPD, data specifically focusing on acutely hospitalized patients remain limited. Most existing studies have been conducted in stable outpatient populations or rehabilitation settings, while patients admitted for acute medical conditions represent a particularly vulnerable subgroup characterized by high short-term morbidity and an increased risk of adverse outcomes following hospital discharge.

Furthermore, comprehensive characterization of patients with COPD during acute hospitalization remains challenging in routine clinical practice. Integrating clinical assessment with BIA may provide additional insights into disease severity and systemic involvement at a critical time point. The use of a single, high-performance multifrequency bioimpedance device applied uniformly across all participants ensures methodological consistency and minimizes variability related to device-specific algorithms.

The aim of the present study was to characterize the clinical and bioimpedance profiles of patients with COPD hospitalized for acute medical conditions and to investigate the associations between bioimpedance-derived parameters, disease severity, and patient comorbidities. We hypothesized that selected bioimpedance-derived parameters would be associated with markers of disease severity and comorbidity burden in this patient population.

## 2. Materials and Methods

### 2.1. Study Design and Setting

We performed a cross-sectional analysis of baseline data from a prospective observational cohort of patients hospitalized with COPD in the Internal Medicine Clinic of the Clinical Emergency Hospital of Bucharest, Romania. The analysis is part of an ongoing prospective observational study conducted in a real-world acute care setting. This study was performed in accordance with the Declaration of Helsinki and was approved by the Scientific Research Ethics Committee of the Clinical Emergency Hospital of Bucharest (approval number 10611, dated 9 December 2024). All participants provided written informed consent prior to enrollment.

COPD was defined based on documented spirometric evidence of persistent airflow limitation, in accordance with established international guidelines. COPD severity was classified using spirometric staging according to the Global Initiative for Chronic Obstructive Lung Disease (GOLD) criteria, based on post-bronchodilator forced expiratory volume in one second (FEV_1_).

### 2.2. Study Population

A total of 72 patients were consecutively enrolled among hospitalized patients who met the predefined eligibility criteria and provided written informed consent. Eligible participants were recruited from patients hospitalized between 9 December 2024 and 17 October 2025.

Patients were eligible for inclusion regardless of the primary reason for hospital admission and were included if they met the following criteria:

- Age of 18 years or older;
- Hospitalization in the Internal Medicine Clinic of the Clinical Emergency Hospital of Bucharest, Romania, during the study period;
- Availability of complete medical information, including sociodemographic, clinical, biological, and imaging data, documented in both the electronic medical record system and the patients’ hospital charts (medical observation sheets);
- A previously documented diagnosis of COPD or a clinical suspicion of COPD raised during hospitalization and subsequently confirmed by spirometry;
- Provision of written informed consent and agreement to participate in the research study.

Patients were excluded if any of the following criteria were present:

- Age below 18 years;
- Absence of written informed consent or refusal to participate in this study;
- Bedridden status;
- Inability to maintain an upright standing position, as required for BIA;
- Severe neurocognitive disorders that could interfere with study participation or data reliability;
- Presence of implantable cardiac electronic devices.

### 2.3. Clinical Assessment and Data Collection

Clinical and demographic data were collected using a standardized data collection protocol. Information was obtained from the patients’ medical observation sheets, the hospital electronic medical record system, and direct patient interviews when feasible.

The following sociodemographic variables were recorded: age, sex, smoking status (current smoker, former smoker, or never smoker), and relevant occupational exposure when available.

Clinical data included vital signs routinely recorded during hospitalization, including systolic and diastolic blood pressure and peripheral oxygen saturation, which were extracted from clinical records. The presence of peripheral edema at admission was also recorded as part of the clinical assessment.

Pulmonary function data were obtained from recent spirometric assessments, when available. COPD severity staging was based on spirometry performed either prior to hospitalization, within a maximum interval of three months, or during the index hospitalization, prior to discharge, once clinical stability was achieved. Spirometric parameters were used to confirm the diagnosis of COPD and to support disease severity classification in accordance with established guidelines.

Comorbidities were systematically assessed based on documented medical history and available clinical records. The overall burden of comorbid conditions was quantified using the Charlson Comorbidity Index (CCI). Particular attention was paid to the presence of cardiovascular comorbidities, given their known impact on prognosis in patients with COPD.

Paraclinical data obtained as part of routine clinical care were extracted from the hospital electronic medical record system and patients’ medical observation sheets. N-terminal pro-B-type natriuretic peptide (NT-proBNP) levels were measured in all patients when the assay was available in the hospital laboratory.

Transthoracic echocardiography was performed when clinically indicated, according to the judgment of the treating physician, as part of routine clinical evaluation. When available, left ventricular ejection fraction (LVEF) values were extracted from echocardiographic reports and included in the analysis.

Functional status and disease severity were evaluated using established clinical instruments. Dyspnea severity was assessed using the modified Medical Research Council (mMRC) dyspnea scale. Symptom burden and disease impact were evaluated using the COPD Assessment Test (CAT). Exercise capacity was evaluated when feasible, using the six-minute walk test (6MWT) performed according to standardized protocols. Composite indices reflecting disease severity, including the BMI, airflow obstruction, dyspnea, and exercise capacity (BODE) index, were calculated when all required components were available.

Anthropometric measurements, including body weight, height, neck circumference, and abdominal circumference, were obtained using standardized methods.

All functional, anthropometric and bioelectrical impedance assessments were performed at the time of hospital discharge. This timing was chosen because, during the acute phase of hospitalization, a substantial proportion of patients were clinically unstable or unable to safely maintain an upright position or reliably complete functional and questionnaire-based assessments. Clinical and demographic data were therefore collected during hospitalization, whereas physical, functional, and bioimpedance measurements were obtained at discharge, prior to leaving the hospital, in order to ensure measurement reliability and reduce potential measurement bias.

### 2.4. Bioelectrical Impedance Analysis

Body composition was assessed using multifrequency BIA (MF-BIA) performed with the InBody 380 device. Measurements were conducted in accordance with the manufacturer’s standardized protocol. Participants were assessed in the standing position, barefoot, with direct contact between the electrodes and the palms and soles, ensuring appropriate positioning and signal acquisition.

The following body composition parameters were obtained directly from the device-generated output sheet of the bioelectrical impedance analyzer:

- Anthropometric parameters: body weight and BMI;
- Adiposity-related parameters: body fat mass (BFM), PBF, and visceral fat level (VFL);
- Hydration-related parameters: total body water (TBW), intracellular water (ICW), extracellular water (ECW), extracellular-to-total body water ratio (ECW/TBW);
- Muscle-related parameters: fat-free mass (FFM), skeletal muscle mass (SMM), and skeletal muscle index (SMI);
- Bioelectrical parameter: phase angle (PhA).

SMI was obtained both as reported by the bioimpedance analyzer and by manual calculation. In the device used, SMI corresponds to appendicular skeletal muscle mass divided by height squared. In addition, SMI was manually calculated as a height-adjusted index using SMM divided by height squared (SMM/height^2^), given the variability in SMI definitions across the literature.

Fat-free mass index (FFMI) was calculated as FFM divided by height squared (FFM/height^2^).

In addition, the extracellular-to-intracellular water ratio (ECW/ICW) was calculated using the ECW and ICW values obtained from the device, and the total body water-to-weight ratio (TBW/weight) was calculated as TBW divided by body weight.

All bioelectrical impedance measurements were performed at the time of hospital discharge, prior to patient release, to ensure clinical stability and patient safety.

### 2.5. Statistical Analysis

Statistical analyses were performed using IBM SPSS Statistics version 22 (IBM Corp., Armonk, NY, USA). Microsoft Excel was used for data management and for the generation of descriptive figures.

Continuous variables were assessed for normality using both the Kolmogorov–Smirnov test and visual inspection of histograms. Visual inspection was additionally used to identify potential outliers and data inconsistencies. Continuous variables are presented as mean ± standard deviation (SD), while categorical and ordinal variables are presented as counts and percentages.

Baseline characteristics of the study population were summarized descriptively and stratified by sex. Subgroup analyses were performed according to sex and clinical characteristics where appropriate. Sex-based analyses were performed given known differences in body composition and bioimpedance parameters between men and women. Comparisons between groups were performed using Student’s *t*-test or Mann–Whitney U test for continuous variables, as appropriate, and the chi-square test or Fisher’s exact test for categorical variables.

Associations between selected bioimpedance-derived parameters and clinical or functional variables were explored using Pearson correlation analysis. To account for potential confounding, partial correlation analyses adjusted for CCI and BODE index were performed.

To further evaluate independent associations, multivariable linear regression analysis was performed with the BODE index as the dependent variable. Age, sex, PhA, and the CCI were included as candidate predictors. A backward elimination approach was applied to identify variables independently associated with the outcome.

Missing data were minimal and were handled by complete-case analysis.

All statistical tests were two-tailed, and a *p*-value < 0.05 was considered statistically significant.

### 2.6. Artificial Intelligence

An artificial intelligence tool (ChatGPT; OpenAI, San Francisco, CA, USA, based on the GPT-5.3 model) was used to assist with language editing and improvement of English phrasing. The authors reviewed and approved all content and take full responsibility for the final manuscript.

## 3. Results

### 3.1. Baseline Characteristics of the Study Population

The baseline demographic, clinical and paraclinical characteristics of the study population are summarized in Table 1, including both overall data and sex-stratified analyses, together with the corresponding *p*-values for between-group comparisons. The study sample consisted predominantly of older adults, with a relatively balanced sex distribution and a slight male predominance (43% females and 57% males).

**Figure 1:**
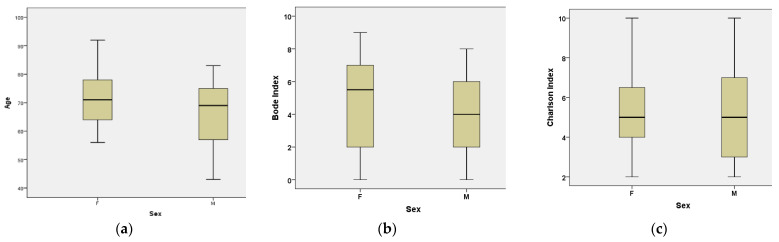
Boxplots of age, BODE index, and CCI according to sex:(**a**) Age; (**b**) BODE index; (**c**) Charlson Comorbidity Index. M—male; F—female.

Within the study sample, the reason for hospital admission differed significantly between sexes. Women also had significantly higher CAT scores compared with men (*p* = 0.029). In addition, women tended to be older and had higher BODE and CCI values (Figure 1); however, these differences did not reach statistical significance.

A similar pattern was observed among patients with occupational exposure to noxious agents, who exhibited higher BODE and CCI scores, although these differences also failed to reach statistical significance (*p* = 0.295 and *p* = 0.209, respectively).

The primary reasons for hospital admission were predominantly pulmonary causes.

**Figure 2:**
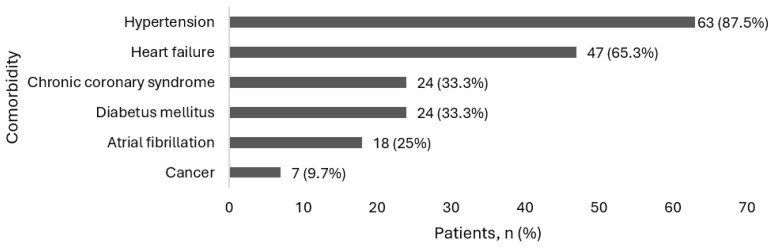
Prevalence of comorbidities in the study population.

Most patients exhibited moderate-to-severe airflow limitation, with a median hospital stay of 8 days. Comorbidity burden, as reflected by the CCI, was considerable, and the distribution of major comorbidities within the study sample is shown in Figure 2.

In sex-based comparisons, chronic coronary syndrome was the only comorbidity that differed significantly between sexes, with a higher prevalence in men (*p* = 0.007).

**Figure 3:**
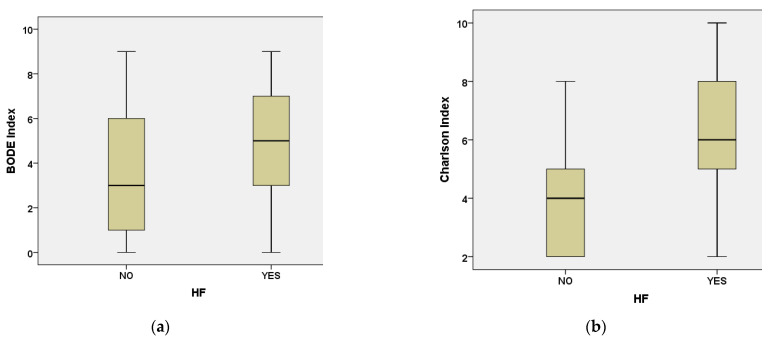
Boxplots of BODE and CCI according to the presence of chronic heart failure. HF—Heart failure. (**a**) BODE index; (**b**) CCI.

Furthermore, BODE and CCI scores were significantly higher in patients with chronic heart failure compared with those without (Mann–Whitney U test, *p* = 0.024 and *p* < 0.001, respectively), as illustrated in Figure 3.

### 3.2. Bioimpedance-Derived Body Composition Parameters

Bioimpedance-derived parameters were grouped into adiposity-related, hydration-related, muscle-related and other indices. Descriptive statistics of these parameters, stratified by sex, are presented in Table 2, along with the corresponding *p*-values for comparisons between women and men.

Several bioimpedance-derived parameters showed significant correlations with clinical, paraclinical or functional variables (Table 3), which represent the main findings of the study.

PhA was significantly associated with markers of COPD severity, cardiovascular burden, and functional status (Table 3). The strongest inverse correlations were observed with the CCI and NT-proBNP levels. Lower PhA values were also associated with a higher BODE index, worse dyspnea scores and presence of edema and heart failure, while a positive correlation was observed with 6MWT. However, in multivariable linear regression analysis using a backward elimination approach, PhA was not an independent predictor of the BODE index. In the final model, only the CCI remained significantly associated with the BODE index (B = 0.380, β = 0.329, *p* = 0.005). These findings suggest that lower PhA values may reflect a higher systemic comorbidity burden and worse functional status.

Regarding hydration-related bioimpedance parameters, Pearson correlation analysis showed no significant correlations with COPD-specific severity markers, but significant associations were observed with the CCI. Both ECW/TBW and ECW/ICW were significantly correlated with the presence of chronic heart failure as a comorbidity. However, this association lost statistical significance after adjustment for CCI in partial correlation analysis, suggesting a potential confounding effect of overall comorbidity burden. The same two parameters were also correlated with LVEF, NT-proBNP levels and the presence of edema at admission.

FFM, SMM and manually calculated SMI (but not device-generated SMI) were significantly correlated with CCI in Pearson correlation analyses (FFM: r = −0.236, *p* = 0.046; SMM: r = −0.277, *p* = 0.019; SMI_manual: r = −0.256, *p* = 0.03).

Furthermore, SMM, SMI (regardless of the calculation method), FFM, and FFMI were significantly correlated with FEV_1_. These associations remained significant after adjustment for CCI and were not substantially altered after further adjustment for both CCI and the BODE index (see Table 4).

Finally, in our study sample, a substantial discrepancy was observed in the diagnosis of obesity depending on the assessment method used. When obesity was defined according to BMI (BMI ≥ 30 kg/m^2^), 47.2% of patients met the diagnostic criteria. In contrast, when excess PBF was used as the defining criterion, 87.5% of patients were classified as obese. The diagnostic concordance between the two methods (PBF vs. BMI) was only 59.7%.

## 4. Discussion

BIA is an accessible and non-invasive method for the assessment of body composition, providing additional information with potential implications for patient management. Its clinical utility continues to be actively investigated. In a previous systematic review, we demonstrated that certain bioimpedance parameters are relevant in the prognostic evaluation of patients with COPD, with the most consistent evidence supporting the role of PhA [10]. Furthermore, several studies have reported associations between BIA-derived parameters and disease severity in COPD [11].

The findings of our study particularly highlight PhA as a bioimpedance parameter associated with COPD severity. In our study sample, PhA was significantly correlated with both the BODE index and the mMRC dyspnea scale in univariate analyses, supporting its relationship with multidimensional disease severity. These results are consistent with previously published data. However, in our study, PhA did not retain independent predictive value in multivariable analysis after adjustment for age, sex, and the CCI. In contrast, the CCI remained independently associated with the BODE index, suggesting that the relationship between PhA and disease severity may be largely influenced by the overall burden of comorbidities. Given that PhA is considered a marker of cellular integrity and function, this finding may indicate that its association with COPD severity reflects, at least in part, underlying systemic and comorbidity-related impairment.

COPD, as a chronic inflammatory disease with progressive evolution, is frequently accompanied over time by multiple chronic conditions, within the complex framework recently described in the 2026 GOLD report as “multimorbidity” [1]. Our study sample reflects this concept, comprising predominantly elderly patients with a substantial comorbidity burden, as indicated by a high mean CCI.

Cardiovascular comorbidities were particularly prevalent, with chronic heart failure present in 60% of patients, hypertension in 87.5%, and atrial fibrillation in 25%. All patients included in this study underwent echocardiographic evaluation and NT-proBNP measurement (within the limits of hospital resource availability), leading to the identification of newly diagnosed heart failure in 25% of patients. These findings underscore the fact that heart failure may remain underrecognized in patients with COPD, likely due to overlapping symptoms, and reinforce the statement from the 2026 GOLD report that “Multimorbidity is often underdiagnosed and undertreated and should be actively searched for in each patient with COPD.” [1].

Our study also identified the bioimpedance parameters ECW/TBW and ECW/ICW as significantly associated with both the presence of heart failure and LVEF. In the literature, an increased ECW/TBW has been linked to a higher risk of COPD exacerbations; however, cardiovascular comorbidities were not taken into account in that analysis [12]. Similarly, ECW/TBW has been reported to correlate positively with symptom burden, although without explicit consideration of underlying cardiac conditions [11].

In contrast, in our study sample, both ECW/TBW and ECW/ICW were associated with cardiac pathology, suggesting that alterations in fluid distribution may reflect cardiovascular involvement rather than being exclusively related to pulmonary disease severity. Therefore, MF-BIA in patients with COPD, including the calculation of ECW/ICW and ECW/TBW, may provide additional information that could prompt further cardiological evaluation. Nevertheless, additional studies are required to clarify the clinical utility of these parameters.

Another important direction of assessment using BIA is the evaluation of muscle mass, given that reduced muscle mass and sarcopenia in patients with COPD have been associated with poorer prognosis and impaired quality of life [1,13].

In our study sample, muscle-related parameters (SMM, SMI, FFM, and FFMI) were significantly correlated with FEV_1_ values, suggesting a relationship between skeletal muscle status and pulmonary function. Importantly, these associations remained significant after adjustment for CCI and persisted following further adjustment for both CCI and the BODE index, indicating that the observed relationships were not solely attributable to comorbidity burden or overall disease severity. This finding may reflect a physiological interplay between skeletal muscle mass and respiratory mechanics, potentially involving inspiratory muscle function; however, this hypothesis requires further investigation.

Obesity is increasingly regarded as a major global health challenge, and growing scientific attention has focused on refining its definition and classification based on the assessment of excess adiposity, which can be evaluated using body composition techniques such as BIA and the measurement of PBF [7].

In our study, a subanalysis revealed a substantial discrepancy in obesity classification depending on the definition applied. While only 47.2% of patients met the traditional BMI-based criterion for obesity (BMI ≥ 30 kg/m^2^), 87.5% exhibited excess adiposity based on PBF and would therefore be classified as obese according to the proposed revised definition.

Both obesity and sarcopenia have been independently associated with adverse clinical outcomes in patients with COPD, and their coexistence may further exacerbate functional impairment [1]. Sarcopenic obesity has been described in patients with COPD and has been associated with impaired pulmonary function and reduced physical performance [14]. In the general population, its prevalence has been estimated at approximately 11% [15], while patients with COPD appear to be at significantly higher risk, being up to three times more likely to present with sarcopenic obesity compared with individuals with normal lung function [16]. Importantly, the definition of sarcopenic obesity remains heterogeneous across studies, which may contribute to variability in reported findings.

From a clinical perspective, the identification of sarcopenic obesity has important implications for patient management [17]. Both components—excess adiposity and reduced muscle mass—represent potentially modifiable targets through integrated lifestyle interventions, including nutritional optimization and structured physical activity. In this context, pulmonary rehabilitation programs, combining aerobic and resistance training, may play a central role in improving exercise capacity and functional status [1]. Nutritional interventions, including adequate protein intake and individualized dietary strategies adapted to associated comorbidities, may further support the preservation or restoration of skeletal muscle mass [17]. Addressing both adiposity and muscle depletion may initiate a positive cycle, in which improvements in body composition contribute to enhanced exercise capacity, while increased physical activity further supports muscle mass restoration and reduction in adiposity.

Although sarcopenia was not formally assessed in our study sample according to current consensus criteria [18], as neither screening with the SARC-F (Strength, Assistance with walking, Rise from a chair, Climb stairs, and Falls) questionnaire nor muscle strength evaluation was performed, the incorporation of functional measures in future research could help better delineate the role of muscle impairment in the multidimensional assessment of COPD.

These findings suggest a complex body composition profile within this study sample and underscore the potential value of bioimpedance assessment in patients with COPD. Such evaluation may facilitate earlier screening for comorbid conditions, particularly cardiovascular disease, and support the timely initiation of primary and secondary preventive measures, as well as tailored interventions including nutritional optimization and structured physical rehabilitation. Nevertheless, further studies are required to better define the clinical implications of these observations.

Several limitations of the present study should be acknowledged. First, this was a single-center study with a relatively limited sample size, which may affect the generalizability of the findings. Second, as this study was conducted in an emergency hospital setting with constrained resources, occasional unavailability of diagnostic kits resulted in missing data for a small number of variables. Finally, the study sample was characterized by a high burden of comorbidities, which may have introduced potential confounding despite adjustment analyses. In addition, an important consideration is that body composition is a dynamic parameter, and hydration-related indices may be subject to short-term variation due to shifts between intracellular and extracellular fluid compartments. In this context, in-hospital therapeutic interventions may have influenced bioimpedance measurements at discharge compared with the time of admission. For example, diuretic therapy may preferentially reduce ECW, potentially leading to lower ECW and ECW/TBW values at discharge, whereas systemic corticosteroids may induce fluid retention through mineralocorticoid effects, depending on the specific agent and administered dose. However, all patients were evaluated at hospital discharge, when they were considered clinically stable. This approach was intended to minimize variability related to the heterogeneity in disease severity and in the reasons for hospital admission at baseline, and to allow more standardized and comparable measurements across the study sample.

**Figure 4:**
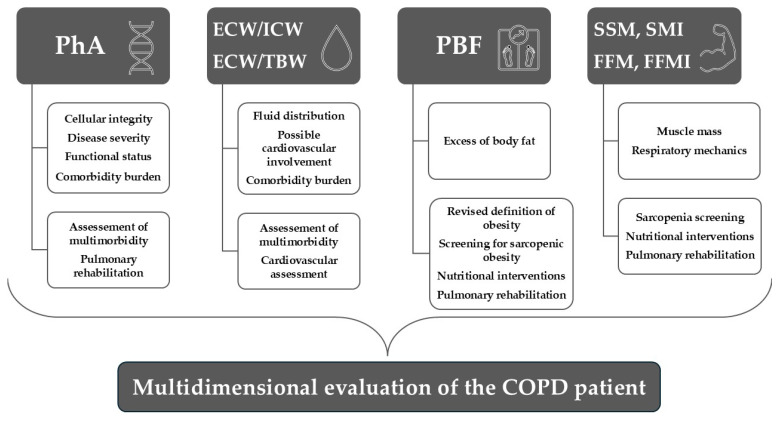
Clinical relevance of MF-BIA-derived parameters in patients with COPD. Upper boxes represent the physiological interpretation of each parameter, while lower boxes summarize potential clinical implications. Abbreviations: ECW/ICW—extracellular-to-intracellular water ratio; ECW/TBW—extracellular-to-total body water ratio; FFM—fat-free mass; FFMI—fat-free mass index; PBF—percentage of body fat; PhA—phase angle; SMI—skeletal muscle index; and SMM—skeletal muscle mass.

Despite these limitations, this study adds to the existing literature by evaluating MF-BIA in a real-world sample of patients with COPD hospitalized in an acute care setting, characterized by multimorbidity and heterogeneous reasons for admission. Importantly, our findings highlight the potential impact of comorbidities—particularly cardiovascular disease—on BIA-derived parameters, underscoring their relevance beyond COPD severity alone. Overall, these results support the added value of MF-BIA in the multidimensional evaluation of patients with COPD, as illustrated in Figure 4.

## 5. Conclusions

Among the BIA-derived parameters, PhA was significantly correlated with both the BODE index and the mMRC dyspnea scale, in line with previously published data. The ECW/TBW and ECW/ICW were associated with the presence of heart failure and with LVEF, suggesting that alterations in fluid distribution may reflect cardiovascular involvement rather than being exclusively related to pulmonary disease severity.

Furthermore, the evaluation of excess body fat may represent a relevant future direction, particularly considering the evolving definition of obesity toward adiposity-based criteria and the discrepancy observed in our study sample between BMI-defined and PBF-defined obesity. In this context, a more refined assessment of sarcopenic obesity—characterized by excess adiposity in the presence of sarcopenia—may be especially relevant in patients with COPD.

Overall, BIA is an accessible and non-invasive investigation with potential value in the multidimensional assessment of complex, multimorbid patients such as those with COPD; however, further studies and methodological standardization are required to clarify its prognostic and clinical utility.

## Figures and Tables

**Table 1:** Baseline characteristics of the study population, overall and stratified by sex.

Characteristic	Total (*n* = 72)	F (*n* = 31)	M (*n* = 41)	p *
Age, years (mean ± SD)	68.4 ± 10.9	70.8 ± 10.2	66.7 ± 11.3	0.115
SpO_2_ at admission, mean ± SD	87.7 ± 7.2	87.6 ± 6.1	87.8 ± 8	0.938
Smoking status				0.552
Current smoker, *n* (%)	31 (43.1)	13 (41.9)	18 (43.9)	
Former smoker, *n* (%)	37 (51.4)	15 (48.4)	22 (53.7)	
Never smoker, *n* (%)	4 (5.6)	3 (9.7)	1 (2.4)	
Pack-years, mean ± SD	48.8 ± 22.8	44.1 ± 18.2 (*n* = 28)	52 ± 25.3 (*n* = 40)	0.159
Occupational exposure, *n* (%)	25 (34.7)	9 (29)	16 (39)	0.265
Length of hospital stay, days, mean ± SD	9.5 ± 6.1	9.9 ± 7.4	9.2 ± 5	0.653
Reason for hospital admission				**0.036**
COPD exacerbation, *n* (%)	25 (34.7)	15 (48.4)	10 (24.4)	
Pneumonia, *n* (%)	19 (26.4)	4 (12.9)	15 (36.6)	
Cardiac, *n* (%)	11 (15.3)	3 (9.7)	8 (19.5)	
Other, *n* (%)	17 (23.6)	9 (29)	8 (19.5)	
CCI, mean ± SD	5.4 ± 2.2	5.5 ± 2.1	5.3 ± 2.3	0.836
GOLD stage, *n* (%)	*n* = 70	*n* = 30	*n* = 40	0.160
1	6 (8.6)	4 (13.3)	2 (5)	
2	31 (44.3)	14 (46.7)	17 (42.5)	
3	23 (32.9)	6 (20)	17 (42.5)	
4	10 (14.3)	6 (20)	4 (10)	
BODE index, mean ± SD	4.4 ± 2.6	4.7 ± 2.9 (*n* = 30)	4.2 ± 2.3 (*n* = 40)	0.432
CAT, mean ± SD	17.2 ± 7.3	19.3 ± 7.2	15.6 ± 7	**0.029**
mMRC, *n* (%)				0.433
0	8 (11.1)	5 (16.1)	3 (7.3)	
1	13 (18.1)	3 (9.7)	10 (24.4)	
2	11 (15.3)	4 (12.9)	7 (17.1)	
3	24 (33.3)	11 (35.5)	13 (31.7)	
4	16 (22.2)	8 (25.8)	8 (19.5)	
FEV_1_ (L), mean ± SD	1.37 ± 0.6 (*n* = 63)	1.1 ± 0.3 (*n* = 24)	1.5 ± 0.6 (*n* = 39)	**0.002**
FEV_1_ (%), mean ± SD	53.8 ± 18.1 (*n* = 65)	58.5 ± 18.5 (*n* = 26)	50.6 ± 17.3 (*n* = 39)	0.082
FEV_1_/FVC, mean ± SD	59 ± 12.3	60 ± 14.7	58.3 ± 10.6	0.595
Comorbidities, *n* (%)				
Hypertension	63 (87.5)	26 (83.9)	37 (90.2)	0.425
Heart failure	47 (65.3)	19 (61.3)	28 (68.3)	0.543
Chronic coronary syndrome	24 (33.3)	5 (16.1)	19 (46.3)	**0.007**
Diabetus mellitus	24 (33.3)	9 (29)	15 (36.6)	0.508
Atrial fibrillation	18 (25)	6 (19.4)	12 (29.3)	0.343
Cancer	7 (9.7)	4 (12.9)	3 (7.3)	0.724

* *p*-values are calculated using the F-test for comparison of means and Fisher’s exact test for comparison of frequencies. Statistically significant *p*-values (*p* < 0.05) are highlighted in bold. Abbreviations: CAT—COPD Assessment Test; CCI—Charlson Comorbidity Index; COPD—chronic obstructive pulmonary disease; F—female; FEV_1_—forced expiratory volume in one second; FVC—forced vital capacity; GOLD—Global Initiative for Chronic Obstructive Lung Disease; M—male; mMRC—modified Medical Research Council dyspnea scale; SD—standard deviation; and SpO_2_—peripheral oxygen saturation.

**Table 2:** Bioimpedance-derived body composition parameters stratified by sex.

Parameters	F	M	p *
Weight (kg), mean ± SD	79.2 ± 27	85.6 ± 20.3	0.252
BMI, mean ± SD	33.4 ± 7.7	29 ± 6.3	**0.029**
PhA, mean ± SD	4.3 ± 0.6	4.5 ± 1	0.287
Adiposity-related parameters			
BFM (kg), mean ± SD	35.8 ± 17.8	28.5 ± 13.3	**0.050**
PBF (%), mean ± SD	42.9 ± 9.8	31.7 ± 10.5	**<0.001**
VFL, mean ± SD	17.6 ± 7.4	14.5 ± 7	0.072
Hydration-related parameters			
TBW (L), mean ± SD	32 ±7.7	42.2 ± 7.6	**<0.001**
ICW (L), mean ± SD	19.3 ± 4.7	25.3 ± 4.7	**<0.001**
ECW (L), mean ± SD	12.6 ± 3	16.8 ± 3.2	**<0.001**
ECW/ICW, mean ± SD	0.655 ± 0.022	0.667 ± 0.055	0.238
ECW/TBW, mean ± SD	0.396 ± 0.008	0.400 ± 0.019	0.271
TBW/weight, mean ± SD	42 ± 7.1	50.5 ± 8.2	**<0.001**
Muscle-related parameters			
FFM (kg), mean ± SD	43.3 ± 10.3	56.4 ± 11.5	**<0.001**
SMM (kg), mean ± SD	23.2 ± 6.2	31 ± 6.1	**<0.001**
SMI_device, mean ± SD	6.7 ± 1.6	8 ± 1.4	**<0.001**
SMI_manual, mean ± SD	9.8 ± 2.1	10.5 ± 1.7	0.108
FFMI, mean ± SD	18.3 ± 3.5	19.1 ± 3.3	0.299

* The *p*-significance refers to F-test (for comparing means) and Fisher’s test (for comparing frequencies). Statistically significant *p*-values (*p* < 0.05) are highlighted in bold. Abbreviations: BFM—body fat mass; BMI—body mass index; ECW—extracellular water; ECW/ICW—extracellular-to-intracellular water ratio; ECW/TBW—extracellular-to-total body water ratio; F—female; FFM—fat-free mass; FFMI—fat-free mass index; ICW—intracellular water; M—male; PBF—percent body fat; PhA—phase angle; SD—standard deviation; SMI_device—skeletal muscle index as provided by the bioimpedance analyzer (appendicular skeletal muscle mass/height^2^); SMI_manual—skeletal muscle index calculated as SMM/height^2^; SMM—skeletal muscle mass; TBW—total body water; TBW/weight—total body water-to-body weight ratio; and VFL—visceral fat level.

**Table 3:** Pearson correlations between bioimpedance parameters and selected clinical variables.

Bioimpedance Parameter	Clinical Variable	r	*p*
PhA	BODE	−0.277	0.02
	mMRC	−0.280	0.017
	CCI	−0.523	<0.001
	Edema	−0.347	0.003
	NT-proBNP	−0.461	0.004
	6MWT	0.322	0.006
	Heart failure	−0.259	0.028
ECW/TBW	CCI	0.394	0.001
	Heart failure	0.305	0.009
	LVEF	−0.272	0.029
ECW/ICW	CCI	0.393	0.001
	Heart failure	0.300	0.011
	LVEF	−0.282	0.023

Abbreviations: BODE—body mass index, airflow obstruction, dyspnea, and exercise capacity index; CCI—Charlson Comorbidity Index; ECW/ICW—extracellular-to-intracellular water ratio; ECW/TBW—extracellular-to-total body water ratio; LVEF—left ventricular ejection fraction; mMRC—modified Medical Research Council dyspnea scale; NT-proBNP—N-terminal pro-B-type natriuretic peptide; PhA—phase angle; r—Pearson correlation coefficient; *p*—*p*-value; and 6MWT—six-minute walk test.

**Table 4:** Pearson and adjusted correlations between bioimpedance parameters and FEV_1_.

Variable	Crude r (*p*)	Adjusted for CCI, r (*p*)	Adjusted for CCI + BODE, r (*p*)
SMM	0.489 (<0.001)	0.434 (<0.001)	0.462 (<0.001)
SMI_device	0.401 (0.001)	0.355 (0.005)	0.387 (0.002)
SMI_calculated	0.332 (0.008)	0.264 (0.038)	0.308 (0.016)
FFM	0.483 (<0.001)	0.438 (<0.001)	0.474 (<0.001)
FFMI	0.310 (0.017)	0.252 (0.048)	0.306 (0.017)

Abbreviations: BODE—body mass index, airflow obstruction, dyspnea, and exercise capacity index; CCI—Charlson Comorbidity Index; FFM—fat-free mass; FFMI—fat-free mass index; SMI_device—skeletal muscle index as provided by the bioimpedance analyzer (appendicular skeletal muscle mass/height^2^); SMI_calculated—skeletal muscle index calculated as SMM/height^2^; SMM—skeletal muscle mass; r—correlation coefficient; and *p*—*p*-value.

## Data Availability

The datasets generated and analyzed during the current study are not publicly available due to patient confidentiality but are available from the corresponding author upon reasonable request.

## Abbreviations

The following abbreviations are used in this manuscript:

6MWT	Six-minute walk test
BFM	Body fat mass
BFMI	Body fat mass index
BIA	Bioelectrical impedance analysis
BMI	Body mass index
BODE	Body mass index, airflow obstruction, dyspnea, and exercise capacity index
CAT	COPD Assessment Test
CCI	Charlson Comorbidity Index
COPD	Chronic obstructive pulmonary disease
DXA	Dual-energy X-ray absorptiometry
ECW	Extracellular water
ECW/ICW	Extracellular-to-intracellular water ratio
ECW/TBW	Extracellular-to-total body water ratio
FEV_1_	Forced expiratory volume in one second
FFM	Fat-free mass
FFMI	Fat-free mass index
FVC	Forced vital capacity
GOLD	Global Initiative for Chronic Obstructive Lung Disease
HF	Heart failure
ICW	Intracellular water
LVEF	Left ventricular ejection fraction
MF-BIA	Multifrequency bioelectrical impedance analysis
mMRC	Modified Medical Research Council dyspnea scale
NT-proBNP	N-terminal pro-B-type natriuretic peptide
PBF	Percentage of body fat
PhA	Phase angle
SMI	Skeletal muscle index
SMM	Skeletal muscle mass
SpO2	Peripheral oxygen saturation
TBW	Total body water
VFL	Visceral fat level
WHO	World Health Organization
